# Assessing Respiratory Motion Stability of Novel ^18^F-Fluorodeoxyglucose Positron Emission Tomography-Derived Morphological Features

**DOI:** 10.3390/diagnostics16070994

**Published:** 2026-03-26

**Authors:** Sze Ian Tan, Kun-Han Lue, Yu-Hung Chen, Sung-Chao Chu, Chih-Bin Lin, Shu-Hsin Liu

**Affiliations:** 1 Department of Nuclear Medicine, Hualien Tzu Chi Hospital, Buddhist Tzu Chi Medical Foundation, Hualien 970473, Taiwan; szeian55555@gmail.com (S.I.T.); kaopectin@yahoo.com.tw (S.-H.L.); 2 Hualien Hsien Association of Radiological Technologists (HHART), Hualien Tzu Chi Hospital, Buddhist Tzu Chi Medical Foundation, Hualien 970473, Taiwan; 3 Department of Medical Imaging and Radiological Sciences, Tzu Chi University, Hualien 970374, Taiwan; john.lue@protonmail.com; 4 School of Medicine, Tzu Chi University, Hualien 970374, Taiwan; oldguy-chu1129@umail.hinet.net; 5 Department of Hematology and Oncology, Hualien Tzu Chi Hospital, Buddhist Tzu Chi Medical Foundation, Hualien 970473, Taiwan; 6 Department of Internal Medicine, Hualien Tzu Chi Hospital, Buddhist Tzu Chi Medical Foundation, Hualien 970473, Taiwan; ferlin@tzuchi.com.tw

**Keywords:** ^18^F-FDG PET/CT, radiomics, respiratory motion, feature stability, lung cancer, prognosis

## Abstract

**Background/Objectives:** Novel hotspot displacement radiomic features (normalized hotspot-to-centroid distance [NHOC]/normalized hotspot-to-perimeter distance [NHOP]) are robust against image resampling and spatial resolution variations. However, their reproducibility under respiratory motion remains unvalidated. This study aimed to evaluate the reproducibility, reliability, and survival prognostic value of NHOC/NHOP features in thoracic ^18^F-fluorodeoxyglucose positron emission tomography/computed tomography (^18^F-FDG PET/CT) images with and without respiratory motion correction and to determine whether these features maintain stability and predictive performance for overall survival (OS) compared with respiratory-stable reference features. **Methods:** We analyzed 138 patients (203 lesions) who underwent ^18^F-FDG PET/CT with and without data-driven respiratory gating. Reproducibility and reliability were assessed using the coefficient of variation (CoV) and intraclass correlation coefficient (ICC), respectively. OS prediction was evaluated using Cox regression and concordance index (c-index) analyses. **Results:** Except for NHOCmax and NHOPpeak, which showed ICC values of 0.782 and 0.93, respectively, the novel morphological features generally exhibited poor reproducibility and moderate reliability (CoV > 20% and ICC < 0.75). In contrast, reference features (entropy-based and sphericity) demonstrated excellent robustness. Motion-corrected NHOCmax showed significant OS prediction for both spatially resampled and non-resampled images. No significant differences in c-indices were observed between motion-corrected and non-corrected features. **Conclusions:** The marked sensitivity of novel hotspot-displacement features to respiratory motion substantially limits their clinical applicability in thoracic disease. To ensure reproducibility and generalizability in future research, prioritizing inherently robust radiomic parameters, such as entropy-based features, is strongly recommended.

## 1. Introduction

^18^F-fluorodeoxyglucose (^18^F-FDG) positron emission tomography (PET) is a cornerstone imaging modality in oncology that provides critical glucose metabolic information for diagnosis, staging, treatment response monitoring, and survival prognosis. The clinical importance of ^18^F-FDG PET was further solidified by the recent Appropriate Use Criteria published in 2025, which underscores its indispensable role in various malignancies, particularly thoracic cancers such as lung and esophageal carcinoma [1,2,3]. Beyond standard visual interpretation and semi-quantitative metrics, such as standardized uptake value (SUV) [4], radiomics has emerged as a transformative field in oncology research. By extracting high-dimensional quantitative features from medical images, radiomics can be used to decode underlying tumor biology and heterogeneity that are imperceptible to the human eye, thereby enhancing prognostic stratification and improving personalized medicine [5,6,7,8].

However, the reliability of PET-based radiomics is frequently challenged by technical factors, with respiratory motion being the primary confounder in thoracic PET imaging. Unlike computed tomography, PET imaging typically requires minutes for each bed position. Therefore, respiratory movement during PET image acquisition causes blurring of the tumor boundaries and smearing of radioactivity distribution, leading to volume overestimation and underestimation of uptake intensity. Crucially, these motion artifacts can considerably alter the extraction of intensity, texture, and shape features [9,10,11]. Recent investigations have demonstrated that many radiomics features are highly sensitive to acquisition conditions, with one study reporting that only a small fraction of features are robust against respiratory motion [5,12]. This lack of reproducibility and reliability limits the clinical generalizability of radiomic signatures and highlights the need for features that remain stable despite respiratory motion.

A recent study introduced a set of novel morphological features based on the normalized distances of metabolic hotspots—maximum standardized uptake value (SUVmax) and peak standardized uptake value (SUVpeak)—to the geometric center and perimeter of the lesion, termed normalized distance from uptake hot spot to tumor centroid (NHOC) and normalized distance from uptake hot spot to tumor perimeter (NHOP), to address challenges in characterizing the intratumoral spatial distribution of glycolysis [13]. These features were designed to quantify the internal spatial displacement of metabolic activity. Preliminary results in a lung cancer cohort suggested that these novel features possess superior robustness against variations in spatial resolution and image resampling compared with traditional texture features [13,14]. However, although their stability against voxel size variations has been documented, their sensitivity to respiratory motion, a critical variable in thoracic oncology, remains unvalidated. It is unclear whether the spatial relationship between metabolic hotspots and tumor geometry remains consistent when the tumor is subjected to the blurring effects of breathing.

In this study, we analyzed a cohort of patients with thoracic hypermetabolic lesions using respiratory-gated (motion-corrected) and non-gated PET images. The primary objective of this study was to evaluate the reproducibility and reliability of novel morphological features under respiratory motion, using well-established respiratory-stable radiomic features as references. The study specifically aimed to determine whether hotspot-displacement metrics maintain stability and predictive performance for overall survival (OS) under respiratory motion and to assess their potential applicability in routine clinical workflows.

## 2. Materials and Methods

### 2.1. Study Population

This retrospective study analyzed 138 patients who underwent clinical ^18^F-FDG PET/computed tomography (CT). The study was approved by the Research Ethics Committee of Hualien Tzu Chi Hospital (protocol ID: IRB110-136-B and IRB114-257-B for later follow-up of lung cancer survival analyses; approval dates: 6 August 2021, and 26 December 2025), and the requirement for informed consent was waived. A total of 203 lesions were included in the quantitative evaluation, comprising 74 patients with biopsy-proven lung cancer and 64 patients with other malignancies. When multiple hypermetabolic lesions were present in a single patient, all measurable lesions meeting the inclusion criteria were selected and delineated independently. Patients were included if their PET/CT images contained at least one measurable hypermetabolic lesion and if both respiratory-gated and non-gated PET data were available for comparison. Lesions were excluded if image quality was insufficient, motion artifacts prevented accurate segmentation, or if the metabolic tumor volume (MTV) was below the minimum voxel requirement for reproducible radiomic extraction of at least 64 voxels.

Clinical data, including age, sex, primary cancer type, and lesion characteristics, were collected from institutional records. This study followed methodology and radiomics acquisition strategies described in the nuclear medicine radiomics literature [15].

### 2.2. ^18^F-FDG PET Imaging Protocol

All patients underwent a standardized ^18^F-FDG PET/CT acquisition protocol. Each patient fasted for 4–6 h before the examination. Blood glucose levels were within clinically acceptable limits (<200 mg/dL) before radiotracer injection.

A fixed radiotracer activity of 400 MBq of ^18^F-FDG was administered intravenously. After injection, patients rested comfortably in a quiet environment for 50–60 min to allow optimal biodistribution and intracellular trapping of ^18^F-FDG. PET/CT was performed using a 4-ring GE Discovery MI system (GE Healthcare, Milwaukee, WI, USA) from the vertex to the mid-thigh. PET data were obtained using a 150 s acquisition per bed position (20 cm/bed). Transmission CT was acquired first with a tube voltage of 120 kV and an automated tube current for attenuation correction and anatomical localization. A data-driven gating (DDG) algorithm was applied for motion correction (Q. Static, GE Healthcare, Milwaukee, USA). Default parameters were applied for all patients in this study, with a phase offset of 30%, a phase window of 50%, and an R-threshold of 15. Both motion-corrected and non-corrected PET images were acquired. PET images were reconstructed using the Bayesian penalized likelihood reconstruction algorithm Q.Clear (β = 550). The matrix size, pixel size, and slice thickness were 256 × 256, 2.73 mm × 2.73 mm, and 2.79 mm, respectively. SUV was calculated and normalized according to body weight and injected radioactivity as follows: SUV = (decay-corrected activity [kBq] per mL of tissue volume)/(injected FDG activity [kBq]/body weight in g).

### 2.3. Image Analysis

#### 2.3.1. Volume-of-Interest (VOI) Delineation

All image analyses were performed using PMOD (version 4.0, PMOD Technologies Ltd., Zurich, Switzerland). For each lesion, an initial VOI was determined manually by a nuclear medicine technologist and subsequently refined using a semi-automated threshold-based contouring approach. This procedure was confirmed by an experienced nuclear medicine physician. An SUV threshold of 40% was used to ensure consistent segmentation. The volume of the segmented mask defined the MTV. SUVmax was automatically identified within the VOI. VOIs for DDG and non-gated PET images were generated independently to avoid misregistration and ensure that feature extraction reflected the inherent properties of each reconstruction. The segmented masks were exported for radiomic feature computation.

#### 2.3.2. Radiomic Feature Extraction

Radiomic analysis was performed using the LIFEx software package (version 7.7.0, IMIV, CEA, Inserm, Orsay, France, www.lifexsoft.org) [16]. Features were extracted with a fixed bin width of 0.25 SUV. Radiomics features, with and without 2 × 2 × 2 mm^3^ voxel spatial resampling, were recorded. Spatial resampling was performed using a B-spline interpolation algorithm [17]. Radiomic feature extraction was performed in compliance with the Image Biomarker Standardization Initiative (IBSI) guidelines [18].

The radiomics analysis focused on two categories of imaging biomarkers. First, four novel morphological features were engineered to quantify the spatial displacement and distribution of metabolic hotspots within the tumor volume.

NHOC represents the normalized spatial separation between the metabolic hotspot and the geometric centroid of the lesion. NHOP quantifies the normalized distance from the hotspot to the nearest point on the tumor boundary. Both metrics were derived using two distinct intensity definitions, maximum intensity voxel (SUVmax) and peak intensity voxel (SUVpeak) [19], yielding four features: NHOCmax, NHOCpeak, NHOPmax, and NHOPpeak. These mathematically describe internal morphological shifts in high-uptake regions and metabolic heterogeneity.

Seven established reference features known for their stability against respiratory motion and image noise were also included. These consist of geometric sphericity (assessing 3D lesion shape) and six high-order texture features: first-order entropy, gray-level run length matrix run entropy, gray-level co-occurrence matrix (GLCM) joint entropy, GLCM sum entropy, and normalized inverse difference metrics (inverse difference normalized [IDN] and inverse difference moment normalized [IDMN]). Reference features served as robust baselines to evaluate the relative performance and stability of the newly proposed morphological indices.

All features were extracted separately from DDG and non-gated images to determine respiratory motion-induced variation.

#### 2.3.3. Data Analysis

All quantitative data preprocessing and statistical analyses were performed using JASP (version 0.19.3.0, University of Amsterdam, Nieuwe Achtergracht 129 B, Amsterdam, The Netherlands) and R (version 4.4.1, R Foundation, Vienna, Austria). Patient demographics are reported in Table 1, and appropriate statistical tests were selected accordingly.

#### 2.3.4. Reproducibility and Reliability Metrics

The reproducibility and reliability of each feature were evaluated using the coefficient of variation (CoV) [5] and intraclass correlation coefficient (ICC) [5], respectively.

Feature stability against respiratory motion was assessed by comparing DDG and non-gated datasets. For the assessment of reproducibility and reliability (CoV and ICC), all 203 lesions were evaluated as independent entities. The CoV was calculated to quantify variability between DDG and non-gated images. Standard interpretative benchmarks were adopted: CoV < 5% indicated excellent stability; 5–10%, 10–20%, and >20% indicated good, moderate, and poor reproducibility, respectively. Reliability was assessed using ICC and classified as excellent (>0.90), good (0.75–0.90), moderate (0.50–0.75), or poor (<0.50). The ICC was computed using a two-way random-effects model with absolute agreement [5]. All stability assessments were performed in the overall cohort and further stratified by MTV (<3 mL vs. ≥3 mL) [20,21]. For the survival analysis in the lung cancer cohort, only the single lesion with the largest metabolic tumor volume (MTV) was selected to represent each patient, ensuring a strict patient-level analysis.

OS was defined as the interval between disease diagnosis and death or censoring at the last follow-up. Associations between PET radiomic features and OS were assessed using Cox regression [22] and Harrell’s concordance index (c-index) [23]. Cox regression results were reported as hazard ratios (HRs) with 95% confidence intervals (CIs). We also computed the 95% CIs for the differences of c-indices between respiratory motion-corrected and non-corrected radiomic features using bootstrap resampling (1000 iterations). Statistical significance was defined as two-sided *p* < 0.05. To account for multiple comparisons in survival analysis, we applied Bonferroni correction. However, considering the high collinearity among several radiomic features (the four entropy-based features and the IDN/IDMN pair, as shown in Figure S1), the significance threshold was adjusted based on the effective number of independent tests (M_eff_ = 8), resulting in a corrected alpha level of 0.00625 (0.05/8).

## 3. Results

### 3.1. Demographics

The baseline demographic and clinical characteristics of the study cohort are summarized in Table 1. The study population consisted of 138 patients, comprising 78 males (56.5%) and 60 females (43.5%), with a mean age of 61.5 ± 11.5 years. A total of 203 hypermetabolic lesions were analyzed using quantitative radiomic analysis. When stratified by lesion size, 143 lesions (70.4%) were classified as large volumes (MTV ≥ 3 mL), whereas 60 lesions (29.6%) were classified as small volumes (MTV < 3 mL). Lung cancer was the predominant primary malignancy, accounting for 53.6% (*n* = 74) of the cohort. The remaining 64 patients (46.4%) had other malignancies, including head and neck (11.6%), colorectal (10.9%), and esophageal cancers (10.9%), which were the most frequent non-lung primary tumors.

### 3.2. Reproducibility and Stability Analysis

Although standard texture and shape features were successfully extracted from all lesions, the extraction of peak-based morphological features (NHOCpeak and NHOPpeak) was limited to a subset of lesions (*n* = 142). This limitation was due to constraints in defining distinct peak foci in lesions with small volumes.

**Figure 1:**
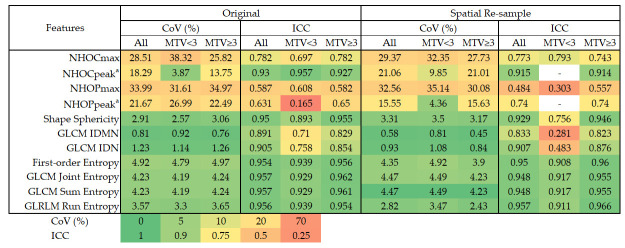
Evaluation of feature reproducibility and reliability across original and spatially resampled datasets, stratified by tumor volume; ^a^ the number of lesions was insufficient for ICC calculation of spatially re-sampled NHOCpeak and NHOPpeak. Abbreviations: NHOC, Novel Hotspot—Occupancy; NHOP, Novel Hotspot—Position; ICC, intraclass correlation coefficient; CoV, coefficient of variation; MTV, metabolic tumor volume; GLCM, gray-level co-occurrence matrix; GLRLM, gray-level run length matrix; IDN, inverse difference normalized; IDMN, inverse difference moment normalized.

The assessment of feature robustness using the ICC and CoV is shown in Figure 1. The seven reference features–sphericity, entropy-based metrics, IDN, and IDMN–demonstrated good to excellent robustness. In contrast, the novel morphological features (NHOC and NHOP families) exhibited less favorable robustness to respiratory motion. Furthermore, NHOCmax, NHOPmax, and NHOPpeak showed suboptimal reliability and reproducibility across both small- and large-volume subgroups.

We also examined the reproducibility and reliability of radiomic features after spatial resampling. Following resampling to isotropic 2 × 2 × 2 mm^3^ voxels, radiomic features showed variable changes in reliability and reproducibility, whereas entropy-based features consistently demonstrated excellent robustness (Figure 1).

### 3.3. Survival Prognostic Values

Patients with lung cancer underwent follow-up for survival analysis. Among the 74 patients with lung cancer, one was lost to follow-up and was excluded, leaving 73 patients for analysis. The estimated median OS was 45.4 months (range: 1.3–113.8 months), and 37 patients (49.3%) died during follow-up. Univariate Cox regression analyses further elucidated the prognostic value of these biomarkers (Table 2). All robust reference radiomic features demonstrated significant prognostic value, with or without respiratory motion correction. Among the reference features, sphericity emerged as a strong protective factor, with higher sphericity significantly associated with prolonged OS (HR = 0.011 and 0.017, *p* < 0.001 for non-corrected and respiratory-corrected sphericity, respectively). Conversely, higher entropy-based features, IDN, and IDMN were significantly associated with an increased risk of shorter OS.

The novel morphological features also showed significant prognostic potential (Table 2 and Table 3), although this was dependent on image processing settings. NHOCmax (HR = 3.644, *p* = 0.017) and NHOCpeak (HR = 4.971, *p* = 0.009) were identified as significant risk factors in the dataset without spatial resampling, indicating that a greater distance between the metabolic hotspot and the tumor centroid was associated with poorer outcomes. However, the significance of these prognostic values varied after spatial resampling (Table 3).

When considering multiple comparisons, the Bonferroni corrected *p*-value was 0.00625. In this regard, sphericity remains statistically significant. Additionally, most of the entropy-based features in the non-spatial resampled dataset remain statistically significant for OS prediction. In spatial re-sampled dataset, the non-corrected entropy and run entropy also exhibit statistical significance.

We also performed multivariable Cox regression analyses. A feature selection process was performed to address the potential redundancy among the imaging features using a correlation matrix (Figure S1). Entropy-based features were highly correlated, and a high correlation coefficient was also observed between IDMN and IDN. Among entropy-based features, we selected run entropy, which showed the most significant prognostic value in univariable analyses, for multivariable analysis. Additionally, IDN was selected because it showed a more significant prognostic value in resampled datasets and non-corrected sdataset without resampling. SUV peak-based novel moprphological features were not included because these features were only available from a subset of lesions. Finally, we did not include NHOPmax owing to the lack of OS prognostic significance in univariable Cox regression analyses. The results of multivariable Cox regression analyses showed that sphericity consistently demonstrated independent prognostic values for OS. The non-corrected run entropy is another independent predictor of OS in the spatially re-sampled dataset (Table S1).

We also compared c-indices between respiratory-corrected and non-corrected features (Table 4). No significant differences in c-indices were found between the two groups.

## 4. Discussion

The primary objective of this study was to bridge the knowledge gap regarding the stability and prognostic utility of novel hotspot-displacement morphological features (NHOC and NHOP) under clinically realistic imaging conditions. Although previous research has established the stability of ^18^F-FDG PET–based novel morphological radiomic features against variations in spatial resolution, their robustness with respect to respiratory motion had not been systematically evaluated prior to clinical application, particularly in thoracic oncology, where lesions are frequently subject to substantial respiratory motion. Compared with well-established respiratory-stable features, our results demonstrated that the novel morphological features were more susceptible to respiratory motion. Although some novel morphological features demonstrated prognostic potential in our cohort, motion sensitivity underscores the need for consistent respiratory gating protocols when comparing results across studies and considering clinical implementation.

Conventional hardware-based respiratory gating typically requires external tracking devices, such as respiratory belts or infrared cameras. However, these conventional methods are often limited by setup complexity, increased scan preparation time, and potential patient discomfort. In contrast, DDG algorithms offer a more feasible and streamlined alternative. DDG extracts respiratory signals directly from the raw PET list-mode data, completely eliminating the need for external tools or additional patient setup. Recognizing this clinical advantage, an increasing number of commercialized PET scanners are being equipped with built-in DDG algorithms. Furthermore, recent studies have successfully utilized DDG to systematically evaluate the effect of respiratory motion on the reproducibility and reliability of PET-derived radiomic features [5], highlighting the practical utility of DDG in advancing motion-robust radiomic research.

Our investigation builds upon the foundational work of Hovhannisyan-Baghdasarian et al. [13], who introduced the NHOC and NHOP metrics as robust surrogates for quantifying the “centrifugal” migration of metabolic hotspots. Their study validated these novel morphological features against variations in reconstruction settings and spatial resolution in a cohort of patients with lung cancer, suggesting a high degree of generalizability [13]. However, respiratory motion is another essential factor that may reduce the reliability and reproducibility of PET-based radiomics features in thoracic lesions. Respiratory motion induces a partial volume effect, altering lesion intensity, volume, shape, and heterogeneity. Recent phantom and patient studies have demonstrated variable susceptibility of PET-based radiomic features to respiratory motion [9,11,17,24,25,26]. Blurring caused by respiratory motion leads to overestimation of tumor boundaries and smearing of internal avidity distributions, directly affecting the spatial coordinates of both lesion centroids and perimeters. Additionally, respiratory motion is not a simple linear translation; it involves complex non-rigid deformation. While the lesion centroid moves with the overall lung excursion, the ‘hotspot’ may shift independently within the blurred volume due to varying regional ventilation and target motion. This asynchronous displacement between the hotspot and the object’s geometric center directly destabilizes the spatial relationship that defines NHOC and NHOP. Consequently, NHOC and NHOP, which depend on these spatial characteristics, are susceptible to respiratory motion. Consistent with this expectation, our results demonstrated that the reliability and reproducibility of these novel features were adversely affected by respiratory motion. Lesion size is another factor influencing respiratory robustness. Although lesions > 3 mL typically exhibit better reliability under respiratory motion [5,25,27,28], in our study, the novel morphological features showed suboptimal reliability and reproducibility across both small- and large-volume subgroups. Furthermore, NHOCpeak and NHOPpeak quantify the relative displacement of the hotspot based on SUVpeak, defined as the average SUV within a 1 mL spherical volume centered on the hottest voxel of the tumor [19,29]. Therefore, NHOCpeak and NHOPpeak rely on sufficient voxel counts for calculation and cannot be computed for small lesions—in our study, 79 of 203 lesions (38.9%) were excluded—further restricting their applicability and clinical utility.

In contrast, entropy-based features (including first-order entropy, joint entropy, sum entropy, and run entropy) and sphericity generally exhibited good to excellent robustness under respiratory motion (CoVs < 5% and ICCs > 0.90). The reliability and reproducibility of these robust features were not substantially influenced by lesion size. These findings are consistent with prior reports [5,25]. In addition, we tested the respiratory motion stability of the radiomic features by incorporating the effect of spatial resampling. The Image Biomarker Standardization Initiative guidelines [18] recommend resampling images to isotropic voxels (such as 2 × 2 × 2 mm^3^) to standardize feature extraction and improve the generalizability of study results. Consequently, an increasing number of radiomic studies have incorporated spatial resampling into their methodologies [8,30,31,32]. After spatial resampling, entropy-based features were minimally affected and maintained excellent reliability and reproducibility, in contrast to the novel morphological features, which consistently exhibited suboptimal respiratory stability. Notably, sphericity, IDN, and IDMN showed substantial reductions in reliability for small lesions. These findings suggest that differences in feature computation formulas may confer variable sensitivities to spatial resampling, resulting in heterogeneous changes in feature stability. Further investigation is warranted to elucidate the underlying mechanisms driving this instability and to determine whether feature-specific correction or selection strategies are necessary when spatial resampling is applied. Collectively, our results suggest that entropy-based features represent more generalizable imaging biomarkers and may be better suited for translational research in thoracic diseases.

In addition to respiratory motion, variations in PET scanners and reconstruction algorithms significantly affect the reproducibility and reliability of PET-derived radiomic features [33,34]. Interestingly, the radiomic features resilient to respiratory motion— such as entropy-based texture features and sphericity—also tend to exhibit high robustness against variations in scanner hardware and reconstruction algorithms [33,34]. This cross-condition stability further underscores the overarching robustness and generalizability of these specific features for multicenter applications. However, it is important to note that our current study was conducted using a single digital PET/CT system with a specific Bayesian penalized likelihood reconstruction algorithm (Q.Clear, β = 550). The robustness of radiomic features across different Q.Clear penalization factors (β values) or between Q.Clear and traditional ordered-subset expectation maximization (OSEM) algorithms has not yet been systematically tested. Therefore, further studies incorporating heterogeneous imaging parameters and reconstruction settings are needed to comprehensively evaluate the generalizability of these novel metrics.

Lung cancer is among the most frequently diagnosed malignancies worldwide. Owing to the lack of early symptoms, most patients present with metastatic disease at diagnosis, resulting in poor survival outcomes. Lung cancer accounts for the highest cancer-related mortality worldwide [35,36,37,38]. Biomarkers for prognostic stratification in lung cancer are essential for precision medicine. Therefore, our study further examined the prognostic value of radiomics features in a subgroup of patients with lung cancer. Some of the novel morphological features demonstrated survival prediction potential (Table 2 and Table 3), consistent with the findings of Hovhannisyan-Baghdasarian et al. [13]. In contrast, respiratory-stable radiomic features demonstrated significant associations with OS (*p* < 0.05) irrespective of motion correction or spatial resampling, reaffirming their robustness. To evaluate the effect of respiratory motion correction on the prognostic value of radiomic features, we compared the c-indices between motion-corrected and non-corrected images. The c-indices were not significantly different between the respiratory motion-corrected and non-corrected images for OS prediction. These findings suggest that, although the novel morphological features exhibit suboptimal reliability and reproducibility under respiratory motion, their associations with survival outcomes remain largely preserved. Therefore, these features may still be valuable for survival prognosis. However, compared with respiratory-stable features, concerns regarding generalizability remain. Studies using these novel features may have limited external validity or require strict standardization of imaging protocols to ensure generalizability in thoracic disease research.

This study has some limitations. First, patients were retrospectively enrolled. Second, only 37 died during follow-up, and the high dimensionality of test variables may limit the robustness our survival analysis. Third, owing to the inherited volume constraints of SUVpeak-based novel morphological features, these features were not available for 38.9% of lesions. This exclusion may introduce selection bias. Finally, the histopathologies of the lung cancer cohorts were heterogeneous, and variations in clinical variables may have affected survival analyses. However, owing to the limited event count, properly adjusting these clinical covariates to identify independent prognosticators would be difficult. Although most radiomic features showed significant prognostic value, further validation in a larger and more uniform lung cancer cohort would be ideal.

## 5. Conclusions

In conclusion, our preliminary study demonstrates that the proposed hotspot-displacement features exhibit only poor to moderate reproducibility and reliability under the influence of respiratory motion. Although they showed some potential for survival prediction, this marked motion sensitivity substantially limits their direct clinical applicability in thoracic lesions. Furthermore, the statistically significant OS prediction of these hotspot-displacement features were lost after spatial re-sampling and were confined to univariate analysis. Consequently, these novel morphological features might only be considered for extrathoracic diseases where respiratory motion is less pronounced. For thoracic oncology applications, extreme caution is warranted when evaluating these metrics, and strictly standardized imaging protocols are prerequisite. To ensure generalizability and reproducibility in future research, prioritizing inherently robust radiomic parameters, such as entropy-based features, is strongly recommended over these motion-sensitive metrics.

## Figures and Tables

**Table 1:** Summary of patient demographics and lesion characteristics included in the analysis (*n* = 138).

Characteristic	Value (%)
Sex, *n*	138
Male	78 (56.5%)
Female	60 (43.5%)
Age	61.5 ± 11.5
Lesion	203
MTV < 3 mL	60 (29.6%)
MTV ≥ 3 mL	143 (70.4%)
Cancer	
Lung cancer	74 (53.6%)
Small cell carcinoma	3 (2.2%)
Adenocarcinoma	61 (44.2%)
Squamous cell carcinoma	9 (6.5%)
Poorly differentiated carcinoma	1 (0.7%)
Others	64 (46.4%)
Head and neck cancer	16 (11.6%)
Colorectal cancer	15 (10.9%)
Esophageal cancer	15 (10.9%)
Breast cancer	6 (4.3%)
Lymphoma	4 (3.0%)
Gynecologic cancer	3 (2.2%)
Genitourinary cancer	2 (1.4%)
Pancreatic cancer	2 (1.4%)
Liver cancer	1 (0.7%)

**Table 2:** Univariable Cox proportional hazards analysis of overall survival in the image dataset without spatial resampling (*n* = 73).

Features	OS			
	MC		NMC	
	HR (95% CI)	*p*-Value	HR (95% CI)	*p*-Value
SUVmax	1.081 (1.018–1.147)	0.010	1.090 (1.023–1.161)	0.007
NHOCmax	2.841 (1.042–7.747)	0.041	3.644 (1.263–10.509)	0.017
NHOCpeak ^a^	4.813 (1.337–17.331)	0.016	4.971 (1.496–16.518)	0.009
NHOPmax	0.152 (0.01–2.329)	0.176	0.119 (0.01–1.432)	0.094
NHOPpeak ^a^	0.020 (6.374 × 10^−4^–0.599)	0.024	0.042 (0.002–0.988)	0.049
Sphericity	0.017 (0.002–0.177)	<0.001	0.011 (8.132 × 10^−4^–0.141)	<0.001
Entropy	1.655 (1.148–2.386)	0.007	1.849 (1.236–2.765)	0.003
Joint Entropy	1.371 (1.097–1.713)	0.006	1.405 (1.121–1.762)	0.003
Sum Entropy	1.371 (1.097–1.713)	0.006	1.405 (1.121–1.762)	0.003
Run Entropy	1.803 (1.196–2.718)	0.005	2.119 (1.335–3.364)	0.001
IDN ^b^	1.011 (1.001–1.022)	0.028	1.013 (1.002–1.024)	0.019
IDMN ^b^	1.019 (1.002–1.037)	0.029	1.023 (1.004–1.042)	0.016

OS, overall survival; MC, motion correction; NMC, non-motion correction; HR, hazard ratio; CI, confidence interval; NHOCmax, normalized distance from hotspot (SUVmax) to centroid; NHOCpeak, normalized distance from SUVpeak to centroid; NHOPmax, normalized distance from hotspot (SUVmax) to perimeter; NHOPpeak, normalized distance from SUVpeak to perimeter; IDN, inverse difference normalized; IDMN, inverse difference moment normalized; SUVmax, maximum standardized uptake value. ^a^ The number of lesions available for NHOCpeak and NHOPpeak analysis was 124 because of the approximately 1 cm^3^ volumetric requirement inherent to the SUVpeak definition. ^b^ The values of IDN and IDMN were multiplied by 1000.

**Table 3:** Univariable Cox proportional hazards analysis of overall survival in the spatially re-sampled image dataset (*n* = 73).

Features	OS			
	MC		NMC	
	HR (95% CI)	*p*-Value	HR (95% CI)	*p*-Value
SUVmax	1.046 (1.011–1.082)	0.009	1.049 (1.012–1.087)	0.009
NHOCmax	2.380 (1.035–5.472)	0.041	1.855 (0.628–5.484)	0.264
NHOCpeak ^a^	3.764 (0.970–14.611)	0.055	5.408 (1.336–21.897)	0.018
NHOPmax	0.475 (0.032–7.060)	0.589	0.530 (0.031–9.150)	0.663
NHOPpeak ^a^	0.052 (0.002–1.495)	0.085	0.319 (0.010–9.895)	0.515
Sphericity	0.010 (6.744 × 10^−4^–0.155)	<0.001	0.004 (2.200 × 10^−4^–0.092)	<0.001
Entropy	1.654 (1.095–2.497)	0.017	1.764 (1.176–2.645)	0.006
Joint Entropy	1.371 (1.053–1.648)	0.016	1.354 (1.086–1.689)	0.007
Sum Entropy	1.371 (1.053–1.648)	0.016	1.354 (1.086–1.689)	0.007
Run Entropy	1.935 (1.187–3.154)	0.008	2.070 (1.273–3.366)	0.003
IDN ^b^	1.016 (1.003–1.030)	0.019	1.014 (1.000–1.028)	0.043
IDMN ^b^	1.035 (1.004–1.067)	0.027	1.029 (0.998–1.061)	0.066

OS, overall survival; MC, motion correction; NMC, non-motion correction; HR, hazard ratio; CI, confidence interval; NHOCmax, normalized distance from hotspot (SUVmax) to centroid; NHOCpeak, normalized distance from SUVpeak to centroid; NHOPmax, normalized distance from hotspot (SUVmax) to perimeter; NHOPpeak, normalized distance from SUVpeak to perimeter; IDN, inverse difference normalized; IDMN, inverse difference moment normalized; SUVmax, maximum standardized uptake value. ^a^ The number of lesions available for NHOCpeak and NHOPpeak was 124 because of the approximately 1 cm^3^ volumetric requirement inherent to the SUVpeak definition. ^b^ The values of IDN and IDMN were multiplied by 1000.

**Table 4:** Comparing c-indices for predicting overall survival between respiratory motion-corrected and non-corrected features (*n* = 73).

**Original**				
**Features**	**c-Index for Non-Corrected PET**	**c-Index for Respiratory Motion Corrected PET**	***p*** **-Value**	**95% CI for c-Index Difference**
SUVmax	0.642	0.643	0.910	−0.020–0.020
NHOCmax	0.613	0.595	0.576	−0.019–0.026
NHOCpeak ^a^	0.600	0.588	0.570	−0.045–0.082
NHOPmax	0.577	0.593	0.740	−0.039–0.064
NHOPpeak ^b^	0.607	0.622	0.738	−0.078–0.110
Sphericity	0.621	0.624	0.719	−0.083–0.116
Entropy	0.640	0.630	0.455	−0.023–0.040
Joint entropy	0.642	0.632	0.305	−0.015–0.035
Sum entropy	0.642	0.632	0.305	−0.015–0.035
Run entropy	0.655	0.635	0.145	−0.045–0.053
IDN	0.607	0.602	0.822	−0.044–0.069
IDMN	0.614	0.601	0.591	−0.009–0.051
**Spatially Re-Sampled Images**				
**Features**	**c-Index for Respiratory Motion-Corrected PET**	**c-Index for Non-Corrected PET**	***p*** **-Value**	**95% CI for c-Index Difference**
SUVmax	0.653	0.641	0.132	−0.034–0.006
NHOCmax	0.553	0.569	0.605	−0.018–0.060
NHOCpeak ^c^	0.602	0.576	0.218	−0.077–0.049
NHOPmax	0.530	0.541	0.797	−0.020–0.076
NHOPpeak ^d^	0.536	0.611	0.077	−0.085–0.098
Sphericity	0.604	0.623	0.272	−0.007–0.163
Entropy	0.631	0.617	0.156	−0.011–0.038
Joint entropy	0.623	0.616	0.550	−0.023–0.033
Sum entropy	0.623	0.616	0.551	−0.023–0.033
Run entropy	0.645	0.623	0.071	−0.073–0.013
IDN	0.606	0.634	0.135	−0.085–0.016
IDMN	0.603	0.636	0.140	−0.006–0.049

PET, positron emission tomography; SUV, standardized uptake value; IDN, inverse difference normalized; IDMN, inverse difference moment normalized; CI, confidence interval. ^a^ This analysis was available for 61 cases. The calculation of NHOCpeak was unsuccessful in 10 patients’ original and respiratory motion-corrected images and in 2 patients’ motion-corrected images. ^b^ This analysis was available for 61 cases. The calculation of NHOPpeak was unsuccessful in 10 patients’ original and respiratory motion-corrected images and unsuccessful in 2 patients’ motion-corrected images. ^c^ This analysis was available for 55 cases. The calculation of NHOCpeak was unsuccessful in 15 patients’ original and respiratory motion-corrected images, one patient’s motion-corrected image, and one patient’s non-corrected image. ^d^ This analysis was available for 55 cases. The calculation of NHOPpeak was unsuccessful in 15 patients’ original and respiratory motion-corrected images, one patient’s motion-corrected image, and another patient’s non-corrected image.

## Data Availability

The original data presented in the study are openly available in FigShare at https://doi.org/10.6084/m9.figshare.31827841, accessed on 22 March 2026.

## Supplementary Materials

The following supporting information can be downloaded at: https://www.mdpi.com/article/10.3390/diagnostics16070994/s1, Figure S1: The correlation matrix of NHOCmax, sphericity, entropy-based features, IDN, and IDMN. Entropy-based features are highly correlated. A high correlation coefficient is also observed between IDMN and IDN. Table S1: The Results of the Multivariable Survival Analysis (*n* = 73).

## Abbreviations

The following abbreviations are used in this manuscript:

^18^F-FDG	^18^F-fluorodeoxyglucose
CI	Coefficient interval
C-index	Concordance index
CoV	Coefficient of variation
DDG	Data-driven gating
GLCM	Gray-level co-occurrence matrix
GLRLM	Gray-level run length matrix
HR	Hazard Ratio
IBSI	Image Biomarker Standardization Initiative
ICC	Intraclass correlation coefficient
IDMN	Inverse difference moment normalized
IDN	Inverse difference normalized
IRB	Institutional Review Board
MC	Motion Correction (respiratory-gated)
MTV	Metabolic Tumor Volume
NHOC	Normalized hotspot-to-centroid distance
NHOP	Normalized hotspot-to-perimeter distance
NMC	Non-motion correction (non-gated)
OS	Overall Survival
OSEM	Ordered-Subset Expectation Maximization
PET	Positron emission tomography
PET/CT	Positron emission tomography/computed tomography
SD	Standard deviation
SUV	Standardized uptake value
SUVmax	Maximum standardized uptake value
SUVpeak	Peak standardized uptake value
VOI	Volume-of-interest
