# Supplementary material for: Assessing Respiratory Motion Stability of Novel 18F-Fluorodeoxyglucose Positron Emission Tomography-Derived Morphological Features
Source: Diagnostics (Basel). 2026 Mar 26;16(7):994. doi: 10.3390/diagnostics16070994 (PMC13073649; doi:10.3390/diagnostics16070994)
Supplement: Supplementary file 1 [file diagnostics-16-00994-s001.zip › diagnostics-4210208-supplementary.pdf]

Supplementary Materials

Assessing respiratory motion stability of novel ^{18}F-FDG PET-derived morphological features

Tan Sze Ian[1,2], Kun-Han Lue[3], Yu-Hung Chen[1,3,4,*], Sung-Chao Chu[4,5], Chih-Bin Lin[6], Shu-Hsin Liu[1]

1 Department of Nuclear Medicine, Hualien Tzu Chi Hospital, Buddhist Tzu Chi Medical Foundation, Hualien 97002, Taiwan); kaopectin@yahoo.com.tw (S.-H.L.); jedimasterchen@hotmail.com (Y.-H.C.); szeian55555@gmail.com (T.S.I)
2 Hualien Hsien Association of Radiological Technologists (HHART)
3 Department of Medical Imaging and Radiological Sciences, Tzu Chi University, Hualien, Taiwan; john.lue@protonmail.com (K.-H.L.)
4 School of Medicine, Tzu Chi University, Hualien, Taiwan
5 Department of Hematology and Oncology, Hualien Tzu Chi Hospital, Buddhist Tzu Chi Medical Foundation, Hualien, Taiwan; oldguy-chu1129@umail.hinet.net (S.-C.C.)
6 Department of Internal Medicine, Hualien Tzu Chi Hospital, Buddhist Tzu Chi Medical Foundation, Hualien, Taiwan ferlin@tzuchi.com.tw (C.-B.L.)
* Correspondence: jedimasterchen@hotmail.com (Y.-H.C.); Tel.: +886-3-856-1825

Figure S1. The correlation matrix of NHOCmax, sphericity, entropy-based features, IDN, and IDMN. Entropy-based features are highly correlated. A high correlation coefficient is also observed between IDMN and IDN.

SUVpeak-based novel features were not included because these features were only available from a subset of lesions. Furthermore, we did not include NHOPmax owing to the lack of OS prognostic significance in univariable Cox regression analyses.

NHOCmax, normalized distance from hotspot (SUVmax) to centroid; NHOCpeak, normalized distance from SUVpeak to centroid; NHOPmax, normalized distance from hotspot (SUVmax) to perimeter; NHOPpeak, normalized distance from SUVpeak to perimeter; IDN, inverse difference normalized; IDMN, inverse difference moment normalized; SUVmax, maximum standardized uptake value;, OS, overall survival.

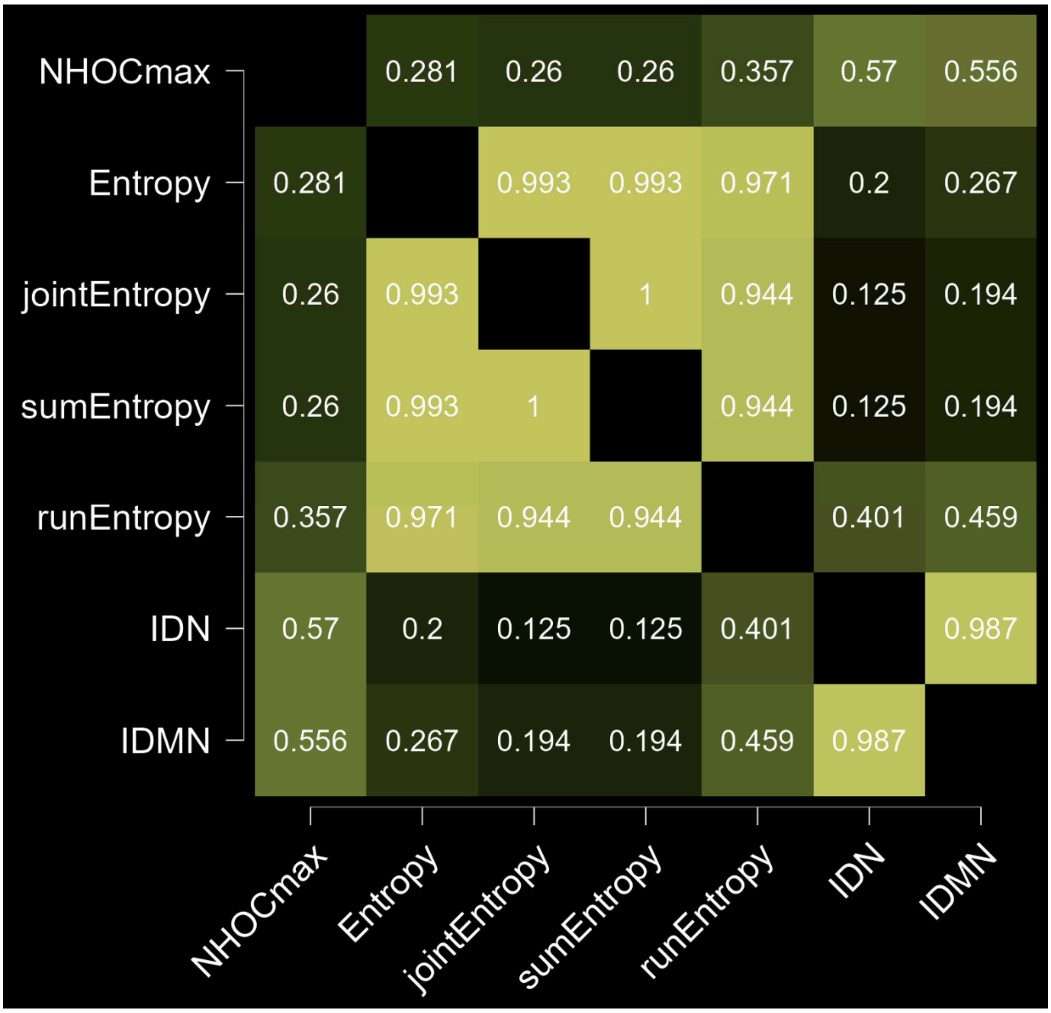

Table S1. The Results of the Multivariable Survival Analysis (n = 73).

Because entropy-based features are high-correlated, we select run entropy, which showed a most significant prognostic value in univariable results, for multivariable analysis. Additionally, IDN showed a more significant prognostic value in resampled datasets and non-corrected dataset without resampling. We select IDN for multivariable analysis instead of IDMN. SUVpeak-based novel moprphological features were not included because these features were only available from a subset of lesions. Furthermore, we did not include NHOPmax owing to the lack of OS prognostic significance in univariable Cox regression analyses.

**Features**	**OS in dataset without spatial re-sampling**			
	**MC**		**NMC**	
	**HR (95% CI)**	***p*-value**	**HR (95% CI)**	***p*-value**
SUVmax	0.961 (0.807–1.144)	0.657	0.895 (0.721–1.111)	0.315
NHOCmax	0.900 (0.251–3.226)	0.872	0.832 (0.185–3.742)	0.811
Sphericity	0.025 (0.001–0.734)	0.032	0.015 (0.000–0.644)	0.028
Run Entropy	2.045 (0.622–6.725)	0.239	3.802 (0.856–16.884)	0.079
IDN[a]	0.999 (0.985–1.013)	0.873	0.998 (0.982–1.014)	0.815

OS, overall survival; MC, motion correction; NMC, non-motion correction; HR, hazard ratio; CI, confidence interval; NHOCmax, normalized distance from hotspot (SUVmax) to centroid; NHOPmax, normalized distance from hotspot (SUVmax) to perimeter; IDN, inverse difference normalized; IDMN, inverse difference moment normalized; SUVmax, maximum standardized uptake value.

[a]The values of IDN were multiplied by 1000.

**Features**	**OS in dataset with spatial re-sampling**			
	**MC**		**NMC**	
	**HR (95% CI)**	***p*-value**	**HR (95% CI)**	***p*-value**
SUVmax	0.965 (0.862–1.081)	0.540	0.922 (0.816–1.042)	0.193
NHOCmax	0.849 (0.283–2.544)	0.770	0.348 (0.082–1.479)	0.153
Sphericity	0.019 (0.001–0.727)	0.033	0.001 (0.000–0.089)	0.002
Run Entropy	2.537 (0.582–11.060)	0.215	4.748 (1.002–22.500)	0.050
IDN[a]	1.006 (0.991–1.021)	0.464	0.999 (0.984–1.015)	0.919

OS, overall survival; MC, motion correction; NMC, non-motion correction; HR, hazard ratio; CI, confidence interval; NHOCmax, normalized distance from hotspot (SUVmax) to centroid; NHOPmax, normalized distance from hotspot (SUVmax) to perimeter; IDN, inverse difference normalized; IDMN, inverse difference moment normalized; SUVmax, maximum standardized uptake value.
[a]The values of IDN were multiplied by 1000.
